# Talon Cusps in Mandibular Incisors: An Unusual Presentation in a Child Patient

**DOI:** 10.5681/joddd.2011.008

**Published:** 2011-03-18

**Authors:** Prasanna Kumar Rao, Shishir Ram Shetty, Rachana V. Prabhu, K.M. Veena, Laxmikanth Chatra, Prashanth Shenai

**Affiliations:** ^1^ Reader, Department of Oral Medicine and Radiology, Yenepoya Dental College, Yenepoya University, Deralakatte, Mangalore, India; ^2^ Assistant Professor, Department of Oral Medicine and Radiology, A.B. Shetty Memorial Institute of Dental Sciences, Nitte University, Deralakatte, Mangalore, India; ^3^ Professor, Department of Oral Medicine and Radiology, Yenepoya Dental College, Yenepoya University, Deralakatte, Mangalore, India; ^4^ Senior Professor and Head, Department of Oral Medicine and Radiology, Yenepoya Dental College, Yenepoya University, Deralakatte, Mangalore, India; ^5^ Senior Professor, Department of Oral Medicine and Radiology, Yenepoya Dental College, Yenepoya University, Deralakatte, Mangalore, India

**Keywords:** Dental anomaly, Eagle’s talon, evagination, mandibular incisors

## Abstract

Talon cusp is a dental anomaly also known as an eagle’s talon. It is an extra cusp on an anterior tooth which arises as a result of evagination on the surface of a crown before calcification has occurred. The exact etiology is unknown. The inci-dence of talon cusp is less than 6%. Commonly involved teeth are maxillary incisors, usually unilateral but in some instanc-es bilateral. The classical radiographical feature of talon cusp is double teeth appearance. The anomaly has been reported to be unusual in the mandibular dentition. This article reports an unusual case of talon cusp of permanent mandibular central incisors.

## Introduction

Talon cusp, also known as eagle’s talon, is a manifestation of dens evaginatus in the anterior teeth.^1^The incidence has been found to range from less than 1% to 6% of the population, in which 55% occur on the permanent maxillary central incisor, and 33% occur on the permanent maxillary lateral incisor.^2^

In a study carried out for a period of three years, the site distribution of talon cusp in a Turkish population was as follows: maxillary right central incisors 30%, maxillary left central incisors 12%, maxillary right lateral incisors 27%, maxillary left lateral incisors 24%, mandibular right central incisor 3%, mandibular left canine 3%.^3^

The frequencies of tooth affected in descending order are central incisors, lateral incisors and canines. The majority of reports about talon cusp show that the permanent dentition has been involved three times more often than the primary dentition. The anomaly has been reported to be unusual in the mandible.^4^

## Case Report

**Figure 1:**
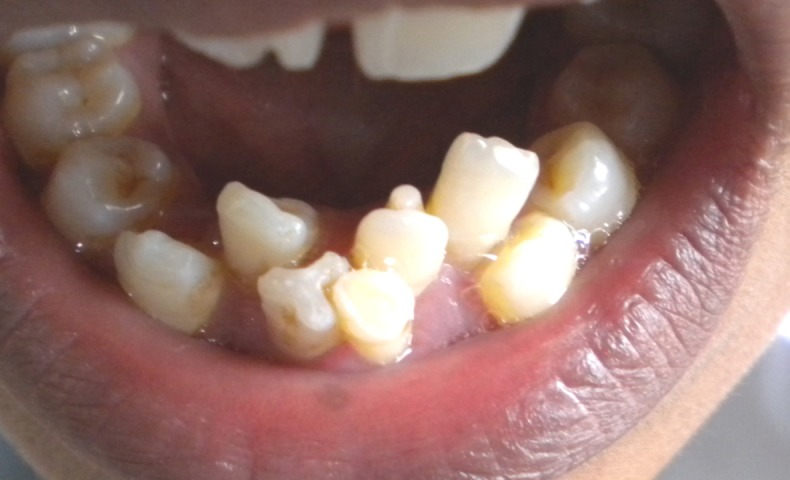
Talon cusp seen as an elevated tubercle in the lingual aspect of mandibular central incisors.

**Figure 2:**
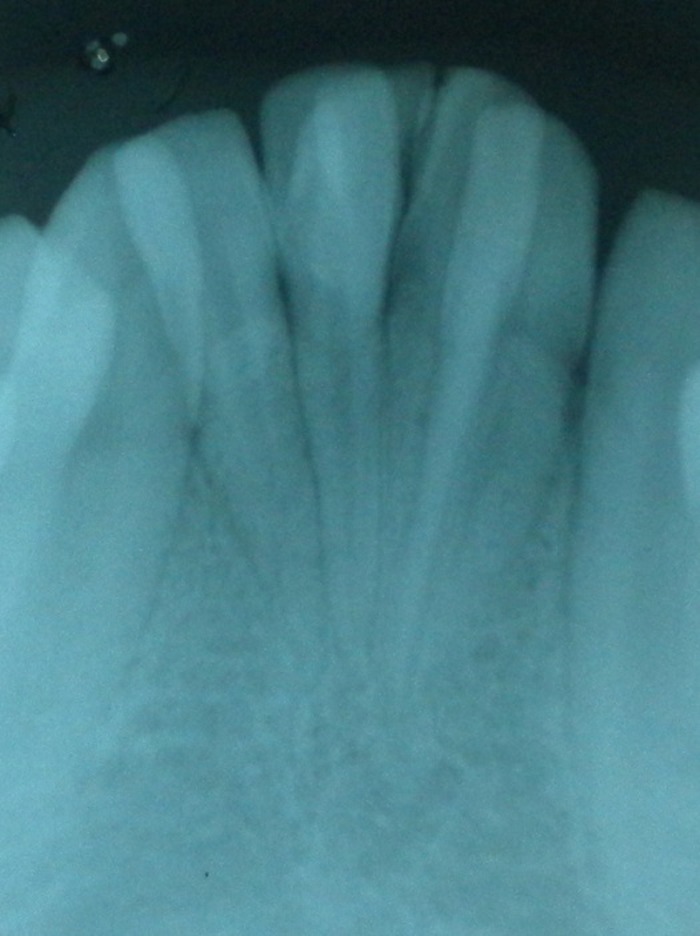
Intra oral periapical radiograph showing double teeth appearance in the mandibular left and right central incisors region.

A 14-year-old boy presented the Department of Oral Medicine and Radiology, Dental Hospital, Mangalore, India, with the complaint of mal-aligned lower anterior teeth. Patient’s medical history was non-contributory, and no abnormalities were noted on general examination. On intra-oral examination, two talon cusps were noted on the lingual surfaces of both mandibular central incisors. An over-retained mandibular primary left central incisor was also observed (Figure 1). On radiographic examination, double-teeth appearance was observed in permanent mandibular right and left central incisors (Figure 2). Because the extra tubercles can cause occlusal interferences and can cause occlusal trauma, the treatment plan called for gradual reduction of the talon cusps on consecutive visits and an application of fluoride at each visit. The patient was later referred for orthodontic correction of mal-aligned teeth.

## Discussion

Talon cusp affects both sexes but males have a higher incidence than females. Most of the cases are unilateral, but one fifth of the cases are bilateral. Studies have revealed that maxillary incisors are most commonly affected and mandibular incisors are least commonly affected.^3^ This abnormality is probably induced by trauma or other localized insults affecting the tooth germ.^5^ Genetic factors have also been suggested by some authors.^1,6,7^ Population-based phylogenic and genetic studies involving dental variations such as cusp of carabelli and talon cusp has been carried out recently in Hungary.^8^ It appears to be more prevalent in patients with Rubinstein-Taybi syndrome, Mohr syndrome (oral-facial-digital syndrome, type-II), Sturge-Weber syndrome (encephalotrigeminal angiomatosis) or incontinentia pigmenti achromians.^9,10^ Our case was not associated with any known abnormal systemic developmental syndromes.

Clinical problems noted with talon cusp cases include attrition, breast-feeding problems, compromised esthetics, occlusal interference, accidental cusp fracture, interference with tongue space, temporomandibular joint pain, displacement of the affected tooth, irritation of tongue during speech and mastication, periodontal problems because of excessive occlusal force, misinterpretation of radiographs of teeth with talon cusp before eruption and caries susceptibility because of developmental grooves on the talon.^11^

Early diagnosis and definitive treatment is important for talon cusp. Caries in the deep developmental grooves on the lateral aspect of the cusp should be removed and the cavity filled with glass ionomer restorative material. Non-carious grooves are cleaned with slurry of pumice, acid etched and sealed with fissure sealant. If talon cusp causes premature contact and occlusal interference, the cusp should be reduced gradually on consecutive visits over 6-8 week intervals to allow time for deposition of reparative dentin for pulpal protection. After each grinding procedure, the tooth surface should be covered with a desensitizing agent. Conservative techniques such as complete reduction of the cusp followed by calcium hydroxide pulpotomy for an immature tooth or root-canal therapy have also been carried out.^12,13^

## Conclusion

Talon cusp although unusually seen, needs to be diagnosed and kept under observation to prevent further complications. Our report highlights this unusual anomaly occurring in an unusual site.
